# Comparison of capillary based microflurometric assay for CD4+ T cell count estimation with dual platform Flow cytometry

**DOI:** 10.1186/1742-6405-3-26

**Published:** 2006-10-16

**Authors:** Madhuri R Thakar, B Kishore Kumar, Bharati A Mahajan, Sanjay M Mehendale, Ramesh S Paranjape

**Affiliations:** 1 National AIDS Research Institute, Bhosari, Pune 411026, India

## Abstract

The CD4+ T cell count estimation is an important monitoring tool for HIV disease progression and efficacy of anti-retroviral treatment (ART). Due to availability of ART at low cost in developing countries, quest for reliable cost effective alternative methods for CD4+ T cell count estimation has gained importance. A simple capillary-based microflurometric assay (EasyCD4 System, Guava Technology) was compared with the conventional flow cytometric assay for estimation of CD4+ T cell counts in 79 HIV infected individuals. CD4+ T cell count estimation by both the assays showed strong correlation (r = 0.938, p < 0.001, 95% CI 0.90 to 0.96). The Bland Altman plot analysis showed that the limits of variation were within agreeable limits of ± 2SD (-161 to 129 cells/mm^3^). The Easy CD4 assay showed 100% sensitivity for estimating the CD4+ T cell counts < 200 cells/mm^3 ^and < 350 cells/mm^3 ^and 97% sensitivity to estimate CD4+ T cell count < 500 cells/mm^3^. The specificity ranged from 82 to 100%. The Kappa factor ranged from 0.735 for the CD4+ T cell counts < 350 cells/mm^3 ^to 0.771 for < 500 cells/mm^3 ^CD4+ T cell counts. The system works with a simple protocol, is easy to maintain and has low running cost. The system is compact and generates minimum amount of waste. Hence the EasyCD4 System could be applied for estimation of CD4+ T cell counts in resource poor settings.

## Background

Three by five initiative by World Health Organization (WHO) has accelerated efforts to provide anti-retroviral treatment (ART) to all those who need it even in the developing world [1]. The ART programme initiated at this scale would require extensive back up for counseling, laboratory investigations to support initiation and monitoring of ART and clinical management of adverse reactions. Important decisions such as when to start anti-retroviral therapy or prophylaxis for opportunistic infections are dependent on the CD4+ T cell count estimation. In the absence of facilities for viral load assays, CD4+ T cell count estimations is being used for monitoring of anti-retroviral therapy [2]. Hence, providing reliable CD4+ T cell counts has become imperative for success of the HIV care and treatment programme.

Flow cytometry has been used as a method of choice for CD4+ T cell measurements since the beginning of HIV epidemic [3,4]. Although flow cytometric estimations give robust and reliable estimations, its high initial and running costs and need for skilled manpower have placed limitations on wider use of flow cytometry in the resource poor settings.

Hence, alternative methods for CD4+ T cell count estimation with lower costs and simplicity in techniques are being explored worldwide [5,6]. The EasyCD4 System is manufactured and marketed by Guava technologies Inc., USA. The system consists of a cell analysis instrument called PCA, a lap top computer with the Guava EasyCD4 software and reagents. The system works on principles of flow cytometry with some modifications. It uses micro capillary as a flow cell unlike the conventional flow cytometry. The whole blood sample is stained with anti-CD3+ (T cell surface marker; present on all T cells) antibodies tagged with phycoerythrin (PE)-Cy5 and anti-CD4+ antibodies tagged with PE. The system uses two-fluorescence parameters (CD3-PE Cy5 and CD4-PE) in combination with forward scatter (FSC) as a measure of relative cell size to analyze the cell population of interest. The CD4+ T cell number is then estimated as the cells simultaneously expressing CD3 and CD4 markers.

We compared the CD4+ T cell estimations in HIV infected individuals using EasyCD4 System with the conventional flow cytometry.

## Results

Thirty-one of the 79 study subjects were females and 48 were males with mean age of 29 and 36 years respectively.

**Figure 1:**
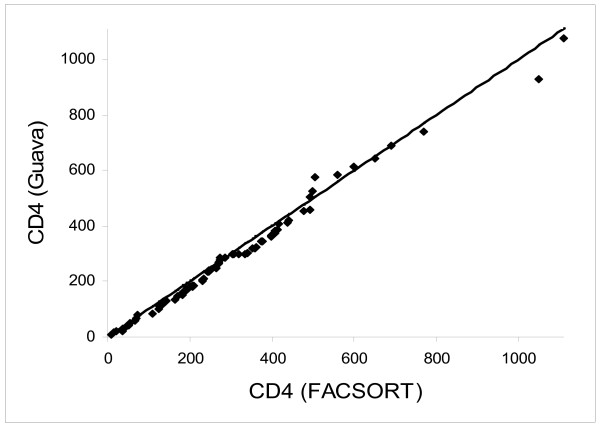
Correlation plot of CD4 cell counts as determined by flowcytometry for CD4+ using dual platform (*X *-axis) and by the EasyCD4 System (Y axis).

The CD4+ T cell counts estimated using EasyCD4 System and flow cytometry showed strong correlation (r = 0.938, p < 0.001, 95% CI 0.90 to 0.96) (figure 1).

The mean absolute count of CD4+ T cells was 306 ± 207 cells/mm^3 ^by the flowcytometry and 290 ± 203 cells/mm^3 ^by the EasyCD4 System. The CD4+ T cell counts estimated in the population under study ranged from 10 to 1110 cells/mm^3 ^by flow cytometer. Hence, the sensitivity and specificity of the Easy CD4 assay was calculated for different categories of CD4+ T cell count as < 200, < 350 and < 500 cells/mm^3^. The Easy CD4 assay showed 100% sensitivity for correct estimation of the CD4+ T cell counts < 200 cells/mm^3 ^and < 350 cells/mm^3 ^and 97% sensitivity to estimate CD4+ T cell count < 500 cells/mm^3^. The specificity ranged from 82 to 100% (Table 1).

**Table 1:** kappa factor and sensitivity and specificity for absolute CD4+ counts determined by EasyCD4 assay.

	N	CD4+ T cell count (cells/mm^3^)				Kappa value	Sensitivity	Specificity
					
		Mean		Median				
		FC*	EasyCD4**	FC*	EasyCD4**			

Total	79	306	290	280	284	---	---	----
CD4 < 500	70	252	227	249	239	0.771	100%	95%
CD4 < 350	52	196	192	210	205	0.735	100%	82%
CD4 < 200	24	108	104	118	112	0.741	97%	100%

*: FC = Flowcytometer

**: Easy CD4: Guava EasyCD4 System

**Figure 2:**
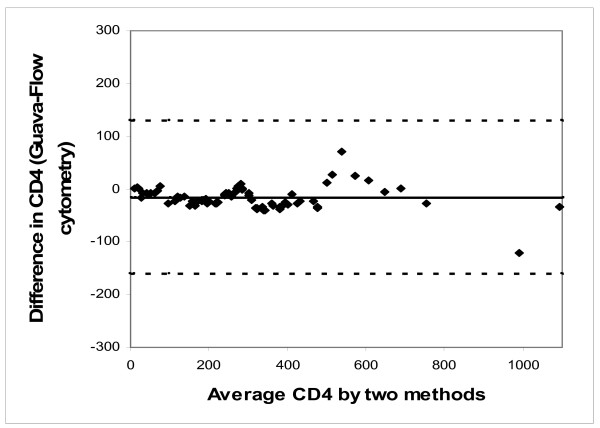
**Bland Altman plot analysis of the CD4+ T cell counts obtained by Easy CD4 assay (Guava) and flow cytometry**. Bland-Altman plot comparing absolute CD4 cell counts estimated by Guava EasyCD4 assay and conventional flowcytometry. The dark continuous line drawn indicates the bias (mean difference), and the dotted lines are the limits of agreement (mean ± 2 SD).

The degree of agreement was estimated by kappa factor (Table 1). The Kappa factor ranged from 0.735 for the CD4+ T cell counts < 350 cells/mm^3 ^to 0.771 for < 500 cells/mm^3 ^CD4+ T cell counts. The Bland-Altman plots also showed that the variation in CD4+ T cell counts between the two methods was within agreeable limits of ± 2 Standard Deviation (SD) (figure 2). The distribution of error was found to be bi-directional.

The comparison between the operational aspects of the two methods is given in Table 2.

**Table 2:** Operational comparison of the two methodologies

**Sr. No.**	**Parameter**	**EasyCD4 System**	**Conventional flow cytometry (FACSort)**
1.	Ease of performance	Excellent	Good
2.	Volume of blood required/test	10 μl	100 μl
3.	Reagents required/test		
	a. Antibodies	1 μl/sample	20 μl/sample
	b. Sheath fluid	---	50 ml/sample
	c. RBC Lysing solution	18 μl/sample	120 μl/sample
4.	Generation of waste/test	200 μl/sample	18 ml/sample
5.	Time required to process one sample	35 minutes	90 minutes
6.	Routine maintenance: Cleaning procedure	Daily (5 minutes) Monthly (-----)	Daily (20 minutes) Monthly (90 minutes)

A limited number of samples (N = 10) were additionally processed by FACSCount (Cat. No.: D0480 Becton Dickinson, USA), a single platform system. The mean CD4 counts obtained by FACSCount and Guava EasyCD4 system were 218 and 221 cells/mm^3 ^respectively. The values obtained by both the methods showed strong correlation (r: 0.98, p < 0.001).

**Figure 3:**
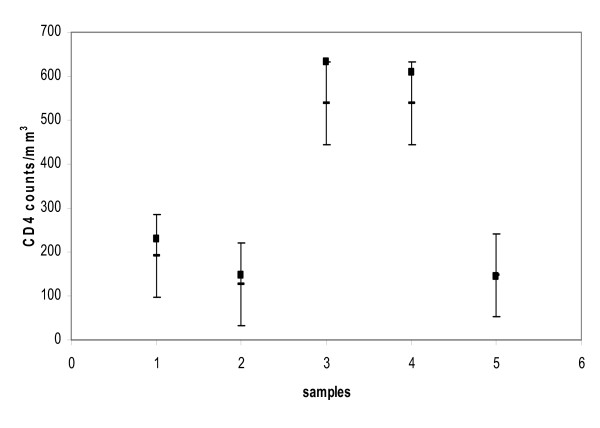
**performance of stabilized blood samples in the guava EasyCD4 assay**. The figure shows performance of stabilized blood samples (samples received for proficiency assessment) in the Guava easy CD4 assay. The error bar shows ± 2 SD of the mean CD4 counts/mm3 (-) for that proficiency run. The CD4 counts obtained by Guava EasyCD4 assay () are between the limit of ± 2SD.

The performance of EasyCD4 assay on five stabilized blood samples showed satisfactory performance within acceptable range of ± 2SD (figure 3).

The intra-sample variation in the EasyCD4 assay was assessed in four samples for the CD4 counts obtained on the same day and after 24 hours. The mean % coefficient of variation (CV) was found to be 6.75% in the range of 1 to 18% while the mean % CV of duplicate testing was found to be 5.17 % (range: 1 to 13%).

## Discussion

Flow cytometry has been accepted as a gold standard for estimating CD4+ T cell count. However, it has high initial as well as running cost and requires frequent maintenance. The frequent power failures, unavailability of temperature controlled laboratories make the maintenance of flow cytometer difficult. Hence the use of flow cytometry is constrained in resource-poor settings. A number of alternative assays have been developed and some are commercially available, i.e., Dynal CD4 assay using magnetic beads and Coulter Cytoshpere assay. These two microscope-based assays were found to be highly comparable with the Flow cytometry [7-9]. However, these alternative assays are fairly labor intensive and, thus less appropriate for testing of a large number of samples. Rodriguez et al developed a microchip-based method for estimation of CD4 percentages at low cost [10]. In addition, modified flow cytometry assays such as a combination of dual platform system and pan-leucogating has shown that reliable estimation of CD4+ T cell count is possible at a reduced cost [11]. Combination of automatic gating with volumetric flow cytometry has been shown to be efficient and gives accurate CD4+ T cell count estimation [12]. The single platform bead based assays available till date were found to be robust, reliable however, expensive.

The EasyCD4 System from Guava Technologies evaluated in the present study is a modified flow cytometer that uses simple volumetric micro capillary-based technology instead of conventional flow cell using sheath fluid for carrying the cells. In conventional flow cytometry, the sheath fluid carries the cells through the LASER path to maintain a single cell suspension so that only one-cell passes through laser beam at one time. In Guava PCA the cells pass through a micro capillary eliminating need of sheath fluid for maintaining single cell suspension. Due to this modification, the volume of accumulated waste is 20 times less than the conventional flow cytometry. Additionally, the conventional flow cytometry in the present study uses the dual side scatter gating depending upon cell morphology (size (FSC) and granularity (SSC)), where as the guava Easy CD4 System uses T cell gating strategy to gate CD3+ cells and estimates CD3+CD4+ cells. The gating on CD3+ T cells removes the monocytes (expressing CD4 but not CD3 on their cell surface) from the gate. The Easy CD4 System is a single platform volumetric system that calculates absolute CD4+ T cell counts on the basis of volume of sample acquired. This eliminates the variability introduced by using Absolute Lymphocyte Count (ALC) from the other analyzer as in dual platform system used in conventional flow cytometry.

The CD4+ T cell counts estimated by EasyCD4 System showed high correlation with the CD4+ T cell counts obtained by conventional flow Cytometry and showed high sensitivity and specificity to identify patients with CD4 + T cell count < 500 cells/mm^3^, < 350 cells/mm^3 ^and < 200 cells/mm^3^. The degree of agreement is high as showed by kappa factor. The Bland-Altman analysis, which is a more reliable method to assess variation, also showed that the variation was within agreeable limits. Hence the method is reliable for making decisions on starting ART or prophylaxis for opportunistic infections. The assay has shown very good agreement with single platform technology like FACSCount or multiTest assay using flowcytometer (13–16) and in the present study although on limited sample size.

The intra-sample %CV in Easy CD4 assay as 6.75% (1 to 18 %), which was comparable with the values (5 to 13%) reported in previous studies (15,16) and less than the %CV reported for "double-platform" systems ranging form 14.5 to 43.4% (mean, 23.4%) (17). Hence, the Easy CD4 assay could be better than the double platform system.

The assay showed satisfactory performance when five stabilized blood samples were assessed for CD4+ T cell counts.

As compared to the conventional flow cytometry the EasyCD4 System was found to be simple to operate, easy to maintain and the equipment requires less space. Since it requires only 10 μl of sample it is possible to explore its application using finger prick blood samples. Hence, it can be adopted for CD4+ T cell count estimation in HIV infected individuals. The running cost of the CD4+ T cell estimation by EasyCD4 System is 5 times lower than the conventional flowcytometry at the current costing. However, the pricing of the reagents and instruments might be subjected to change due to higher demand and wider choice of technologies available to the customer.

EasyCD4 assay, although operationally simple, the users need training in gating of the CD3+ T lymphocytes. The use of minimum quantity of antibodies (1 μl of antibody cocktail) requires precision in technique of reverse pipetting used in case of minute quantities of reagents using the air displacement pipettes as described (18). Also, the EasyCD4 System does not prescribe validity criteria for assessing the formation of the gate. This was found to be extremely critical for reliable gating for accurate estimation of CD4+ T cell counts, to minimize the variations in CD3+ T cell counts and to overcome the acquisition of debris causing high event rate. The laboratory using the equipment needs to set up such criteria locally such as use of commercially available controls or use of healthy individuals sample for gating the CD3+ T cells. This essentially highlights the need of development of laboratory based quality control check on the equipment.

## Conclusion

In conclusion, the availability of EasyCD4 System enhances the options for reliable and valid CD4+ T cell count estimation technology for HIV infected individuals. The validation of the system on finger prick samples could be taken up to assess the simplicity of sample collection and cost reduction.

## Methods

### Study subjects and CD4+ T cell count estimation

79 HIV infected individuals attending the referral clinic of National AIDS Research Institute (NARI), Pune were enrolled in the study after obtaining written informed consent from 5 February to 21 April 2004. The blood sample was collected in a vacutainer containing K3 EDTA to avoid clotting of blood.

The CD4 + T cell counts were estimated by dual platform Flow cytometry (FACSORT, BD 206, Becton Dickinson, USA) as a part of routine investigations using IMK Plus kit (Cat # 349217, BD, USA). The kit included a panel of monoclonal antibodies of CD45-Fluorescein Isothiocynate (FITC)/CD14-Phycoerythrin (PE), CD3-FITC/CD19-PE, CD4-FITC/CD8-PE, CD3-FITC/CD3 HLA-DR-PE and CD3-FITC/CD16+56-PE and SimulSET software. Hundred μl of noncoagulated blood was stained with 20 μl of each of the antibody pair. After 30 minutes incubation the red blood cells were lysed using freshly diluted (1:10) FACS lysing solution (Cat. No.: 349202, Becton Dickinson, USA). The samples were then acquired in the instrument and the lymphocytes were gated on the basis of size (Forward scatter: FSC) and relative granularity (Side scatter: SSC) for further analysis. Two-colour analysis for each antibody subset was performed to obtain percentages of each lymphocyte subset. The run was considered valid only if more than 95% lymphocytes were in the gate.

The absolute CD4+ T cell counts were computed by feeding the ALC in the SimulSET software and were expressed as cells/mm^3^. These ALCs were obtained on the hematology analyzer (Sysmex, Kx21).

### CD4+ T cell count estimation by EasyCD4 System

An aliquot of the same blood samples were coded to blind the technician and processed by Guava Easy CD4 assay (Guava Technologies, USA) on the same day of sample collection. The coding was carried out only when at least three samples were available on a given day to ensure blinding of the laboratory technician.

**Figure 4:**
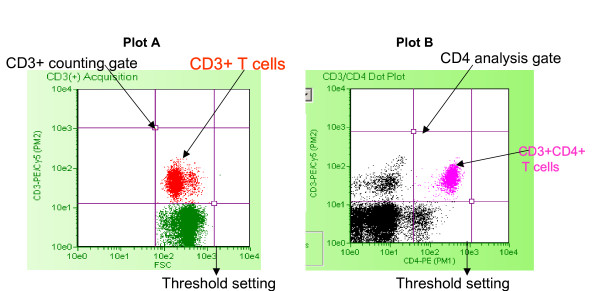
**Scatter plots showing CD3+ T cell gating and CD3+CD4+ T cells in EasyCD4 System**. Plot A: CD3+ cells (in red) are gated using the size (FSC: X axis) and the CD3-PECy5 staining (PM2: Y axis) using the threshold setting markers. Plot B: CD3+CD4+ T cells are gated in CD4 analysis gate using two-color fluorescence CD4- PE (PM1: X axis) and CD3- PECy5 (PM2: Y axis) using the threshold setting markers.

Ten μl of noncoagulated blood was stained with 1:10 diluted antibody cocktail of CD3-PE-Cy5 and CD4-PE or CD3-PE-Cy5 and CD8-PE separately for 15 minutes at room temperature in dark and the RBCs were lysed using the freshly prepared lysing solution for 15 minutes. The instrument was calibrated daily using Guava check beads (cat# 42000070) in triplicate and the instrument was considered to be ready for use if the average CV% for FSC intensity and PM1 and PM2 mean fluorescence Index (MFI) of all three replicates is within 1–5. The sample was acquired within five hours of staining using Cytosoft software. The gate was automatically set around the CD3+ T lymphocytes using the scatter plot showing cells stained with antibodies against CD3 conjugated to PECy5 and the size of the cells as shown in figure 4; plot A. The CD3+CD4 + T cells were further gated using two-fluorescence scatter plot of CD3-PECy5 and CD4-PE (Figure 4; plot B). The Cytosoft software calculates the absolute CD4+ T cell count from total number of cells within the CD4 analysis gate (tCD4), volume of the sample taken up during data acquisition (v) and the dilution factor (df) using the following formula,

Absolute CD4+ T cell count/mm^3 ^= (tCD4 × df)/v cells.

During the preliminary experiments, it was observed that in few cases there was variation in the CD3+ T cell counts obtained in both, sample tubes estimating CD4+ and CD8+ T cell counts and also there was variations in the event rate (no of cells acquired per second). The high event rate was found to occur in case of the samples having a high proportion of non-lymphocyte population in the sample. So the run was considered valid if the variation between the CD3+ T cell counts in both sample tubes (used to estimate CD4 and CD8 T cell counts) was less than 10%, the event rate of acquisition of the sample was less than 700/μl and the CD3+ cells acquired in the gate ranged from 1000 to 2000 cells/μl.

### Data analysis

At the end of the study, the CD4+ T cell counts obtained by the EasyCD4 System were decoded and compared with the CD4+ T cell counts obtained by the flowcytometry. The correlation between the two methods was assessed using Pearson's correlation test and the degree of agreement was estimated by calculating the kappa factor. The Bland-Altman plots were generated for assessment of the variation between the two methods. The sensitivity and specificity of the Easy CD4 assay was estimated for different ranges of CD4+ T cell counts. Data analysis was carried out using SPSS statistical package (version 12.0) to calculate degree of agreement, and to calculate correlation coefficient. The percent coefficient of variation (%CV) was calculated to assess intra-sample variation by using Microsoft Excel software.

## Competing interests

The author(s) declare that they have no competing interests.

## Authors' contributions

MT conceived of the study, and participated in its design, coordination and drafted the manuscript. BM carried out the CD4+ T cell count estimation assays. BK participated in the design of the study and performed the statistical analysis. SM and RP participated in design of the study, monitored the progress and reviewed the draft of the manuscript. All authors read and approved the final manuscript.
